# Pepticombisomes: Biomimetic Vesicles Crafted From Recombinant Supercharged Polypeptides with Uniformly Distributed Side‐Chains

**DOI:** 10.1002/advs.202411497

**Published:** 2025-02-22

**Authors:** Dominik Söder, Melina Schadt, Vladislav S. Petrovskii, Tamás Haraszti, Khosrow Rahimi, Igor I. Potemkin, Nina Yu. Kostina, Cesar Rodriguez‐Emmenegger, Andreas Herrmann

**Affiliations:** ^1^ Institute of Technical and Macromolecular Chemistry RWTH Aachen University Worringerweg 2 52074 Aachen Germany; ^2^ DWI ‐ Leibniz Institute for Interactive Materials Forckenbeckstraße 50 52074 Aachen Germany; ^3^ Physics Department Lomonosov Moscow State University Leninskie Gory 1–2 Moscow 119991 Russian Federation; ^4^ Institute for Bioengineering of Catalonia (IBEC) Carrer de Baldiri Reixac, 10, 12 Barcelona 08028 Spain; ^5^ Institució Catalana de Recerca i Estudis Avançats (ICREA) Passeig Lluís Companys 23 Barcelona 08010 Spain; ^6^ Biomedical Research Networking Center in Bioengineering Biomaterials and Nanomedicine The Institute of Health Carlos III Av. Monforte deLemos 3–5 Madrid 28029 Spain

**Keywords:** biomimetic synthesis, bottom‐up synthetic biology, supercharged peptides, synthetic cells, vesicles

## Abstract

Cell membranes play a key role in bottom‐up synthetic biology, as they enable interaction control, transport, and other essential functions. These ultra‐thin, flexible, yet stable structures form through the self‐assembly of lipids and proteins. While liposomes are common mimics, their synthetic membranes often fail to replicate natural properties due to poor structural control. To address this, pepticombs are introduced, a new family of supramolecular building blocks. They are synthesized by regularly appending anionic surfactants with lipid‐long alkyl tails to cationic amino acid residues of recombinant elastin‐like supercharged unfolded polypeptides (SUPs). Using microscopy techniques and molecular dynamics simulations, the formation of giant unilamellar vesicles, termed pepticombisomes, is demonstrated and their membrane properties are characterized. The molecular topology of pepticombs allows for precise mimicry of membrane thickness and flexibility, beyond classic polymersomes. Unlike the previously introduced ionically‐linked comb polymers, all pepticombs exhibit a uniform degree of polymerization, composition, sequence, and spontaneous curvature. This uniformity ensures consistent hydrophobic tail distribution, facilitating intermolecular hydrogen bonding within the backbone. This generates elastic heterogeneities and the concomitant formation of non‐icosahedral faceted vesicles, as previously predicted. Additionally, pepticombisomes can incorporate functional lipids, enhancing design flexibility.

## Graphical abstract

Pepticombisomes are biomimetic vesicles formed by elastin‐like supercharged unfolded polypeptides (SUPs) uniformly functionalized with anionic surfactants with lipid‐like hydrophobic tails. The molecular design of these supramolecular combs enables precise assembly into membranes with controlled thickness and flexibility. Combining electrostatic interactions and tailored molecular architecture, pepticombisomes mimic natural membranes while offering functionalization potential for advanced applications in synthetic biology and drug delivery.

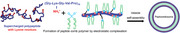

## 1 Introduction

The inherent complexity of natural cells inspires synthetic biology to create minimalistic cell analogs from well‐characterized abiotic constituent parts (bottom‐up approach), bypassing redundancies and mimicking essential cellular functions.^[^ ^1^ ^]^ These cell surrogates offer insights into fundamental processes and open possibilities for systems with augmented functions that surpass natural limits. The cell membrane is an essential component of the cell for which our understanding of its structure and function is still at its infancy. It is formed by the self‐assembly of lipids and proteins and provides integrity to the cellular environment and its compartments.^[^ ^2^ ^]^ Not only does it support complex shapes and provides stability to a cell, but it also supports the cell metabolism,^[^ ^3^ ^]^ cell motion,^[^ ^4^ ^]^ as well as signal transduction and communication.^[^ ^5^ ^]^ This multiplicity of functions is based on the lateral organization of membrane lipids, proteins, and cholesterol into surface topologies (rafts) controlled by non‐covalent interactions.^[^ ^6^ ^]^ Due to this complexity, current membrane model systems aim at recapitulating a limited set of functions within a minimalistic system.^[^ ^1d^ ^]^ Here, the challenge is to design new constituent parts, or building blocks, that can not only faithfully mimic the biophysical properties, but also enable the unparalleled functionality of natural membranes.

Lipids have been the most common choice for constructing synthetic cell membranes, given their prominence as the primary component in natural membranes (≈50 wt.%).^[^ ^7^ ^]^ These liposomes display similar membrane flexibility and lateral mobility,^[^ ^8^ ^]^ while allowing the functionalization with membrane proteins.^[^ ^9^ ^]^ A notable advancement in this area involved the establishment of pathways for artificial lipid synthesis,^[^ ^10^ ^]^ enabling the design of lipid compositions and functionalities previously unattainable in nature.^[^ ^11^ ^]^ However, single‐component liposomes often lack essential membrane constituents, which can be detrimental to maintaining structural integrity. Moreover, many lipids are susceptible to instability either due to the oxidation of double bonds and hydrolysis of ester bonds or the cohesive interactions are insufficient to maintain good mechanical and thermal stability.^[^ ^12^ ^]^ Without mechanisms to replenish or repair degraded lipids, even minor alterations in their structure, such as those caused by oxidation or hydrolysis, significantly impact the overall properties of the liposomes,^[^ ^13^ ^]^ including overall shape, membrane stability, and permeability. This results in breakage, leakage of the content, and uncontrolled aggregation and fusion.^[^ ^12^ ^]^

The challenges with stability could be mitigated by introducing macromolecular amphiphiles where the entropic component further favors stability.^[^ ^14^ ^]^ Thereby, the hydrophobic moieties entangle to form the hydrophobic central region of the membrane, with the hydrophilic parts flanking it, generating the internal and external interface.^[^ ^15^ ^]^ The design of amphiphilic block copolymers (BCPs) allowed for precise adjustment of membrane thickness and flexibility,^[^ ^16^ ^]^ while retaining high thermal and mechanical stability.^[^ ^14^, ^17^ ^]^ They also offer many opportunities for peripheral functionalization.^[^ ^6^, ^18^ ^]^ However, despite the inherent advantages over liposomes, the entanglement of the hydrophobic domain comes at the expense of reduced membrane dynamics and a high membrane thickness associated with the molecular weight of BCPs.^[^ ^17^ ^]^ Moreover, the molecular arrangement greatly hampers the incorporation of membrane proteins, co‐assembly with functional lipids, and the emulation of cellular functions such as exo‐ and endocytosis, fusion, and division.^[^ ^14^, ^16^, ^17^, ^18^, ^19^ ^]^

Several drawbacks have been addressed through the careful molecular design of macromolecules to steer their assembly into more biomimetic vesicles. The two most prominent examples are amphiphilic graft copolymers and Janus dendrimers. The former are built from flexible, low molecular weight poly(dimethyl siloxane) backbones with sparsely grafted poly(ethylene oxide) side chains.^[^ ^20^ ^]^ Graft copolymers drastically reduce the entanglement in the hydrophobic domain and have enabled the incorporation of membrane proteins and the co‐assembly with lipids.^[^ ^20d–f^ ^]^ However, their mixtures with lipids can become unstable and phase separation occurs into nano‐ and microdomains when the molar ratio of lipids exceeds a certain threshold.^[^ ^21^ ^]^ This led to instabilities manifested as division into liposome and polymersome daughter vesicles.^[^ ^20b^ ^]^ The second family of amphiphiles, Janus dendrimers, consists of hydrophilic and hydrophobic dendrons attached to opposite sides of a core. In water, they self‐assemble into vesicles referred to as dendrimersomes.^[^ ^22^ ^]^ As xenobiotic substitutes for lipids and glycolipids, their membranes show biophysical properties comparable to natural cell membranes with equivalent or even greater stability.^[^ ^22^, ^23^ ^]^ Furthermore, they can be mixed with lipids, can harbor proteins, drugs, or nucleic acids, and can perform a rudimentary form of artificial endocytosis.^[^ ^24^ ^]^ They even showed potential for the delivery of RNA.^[^ ^25^ ^]^ Their success is rooted in the precise control of their amphiphilicity and topology but requires substantial synthetic efforts. In this work, we present a new family of vesicles assembled from elastin‐like cationically supercharged unfolded polypeptides to which lipid‐like hydrophobic tails are appended. This concept is inspired by our recent work on i‐combisomes self‐assembled from ionically‐linked comb polymers (iCPs) featuring a hydrophilic poly(carboxybetaine acrylamide‐co‐*N*,*N*‐dimethylamino‐propyl acrylamide) backbone and didodecyl phosphate (DDP) side chains.^[^ ^26^ ^]^

**Figure 1:**
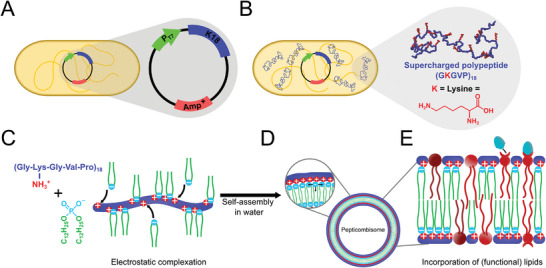
A) Schematic representation of the SUP expression vector in genetically engineered E. coli. B) Schematic representation of the expression of cationic SUP K18. The K18 peptide chain model depicts the 18 pentapeptide repeating units with highlighted lysine residue (red). C) The lysine's free amine confers a net positive charge under physiological conditions and can be complexed with DDP through ionic coupling to form a polyelectrolyte‐surfactant complex (pepticomb). D) The pepticombs self‐assemble in water into pepticombisome vesicles. The DDP residues form the hydrophobic domain. E) The biomimicry of pepticombisomes facilitates the incorporation of lipids of various alkyl chain lengths, allowing surface functionalization through appropriately modified lipids.

Here, the polyacrylamide backbone is replaced with SUPs featuring 18 positively charged pentapeptide units. We refer to this new class of polyelectrolyte‐surfactant complex as pepticombs, as they resemble bottlebrush copolymer structures induced by electrostatic interactions (**Figure** **1**C).^[^ ^27^ ^]^ The salient difference between the iCPs and pepticombs is in the precision of their sequence. iCPs are synthesized by radical copolymerization, an inherently stochastic process resulting in random heteropolymers.^[^ ^28^ ^]^ In stark contrast, the expression of recombinant proteins yields monodisperse biopolymers with a perfectly determined degree of polymerization and amino acid sequence as strictly dictated by the cognate gene,^[^ ^29^ ^]^ setting them apart from conventional synthetic polymers.^[^ ^30^ ^]^ This precise molecular composition ensures that every molecule in a given sample has exactly the same degree of polymerization, composition, topology, and sequence. Consequently, all molecules display the same packing parameter and spontaneous curvature.

Using a combination of optical, electron, and force microscopy techniques, along with atomistic molecular dynamic simulations, we demonstrated the formation of unilamellar vesicles, called pepticombisomes, and characterized their membrane properties. We showed that the pepticombisomes exhibit a biomimetic bilayer thickness. Molecular dynamics simulations revealed that the hydrophobic tails are uniformly distributed along the backbone, assembling into a bilayer of DDP flanked by SUPs in a coiled conformation. Furthermore, the non‐covalent coupling of the hydrophilic and hydrophobic components and the non‐entangled nature of the hydrophobic domains endow these membranes with exceptional flexibility, surpassing even that of liposomes. Interestingly, the ability of the backbone to form intermolecular hydrogen bonds allows for the presence of vesicles with faceted regions without the hydrophobic part being in a gel phase. Lastly, their biomimetic characteristics empower these membranes to facilitate peripheral functionalization through co‐assembly with functional lipid molecules. It is important to emphasize that pepticombisomes are not intended to compete with vesicles produced by cells, which possess not only optimal physical membrane properties but also functional receptors, cytosolic content, and in some cases genetic material. Instead, our goal is to introduce a fundamentally new concept for synthetic cell membrane mimics that captures the main positive features of lipid‐ and block‐copolymer‐based vesicles but adds the prospects of higher levels of programmability of nano and mesoscopic properties as seen in the pioneering and well‐established peptide amphiphile materials. We envision these biomimetic vesicles having a broad impact beyond the field of synthetic cells by enabling the precise design of peptides to introduce lateral interactions to program non‐spherical shapes, encode information, facilitate recognition, or form hybrids with DNA/RNA aptamers.

## 2 Results and Discussion

**Figure 2:**
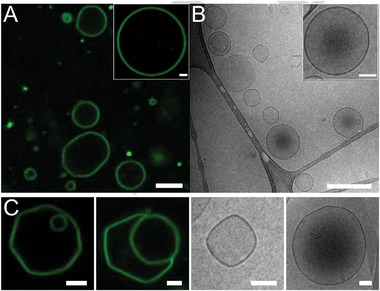
Pepticombisomes as obtained by self‐assembly through thin film rehydration. The formation of predominantly unilamellar vesicles was observed in A) CLSM and B) cryo‐TEM. C) Whereas most vesicles are spherical, some vesicles were observed to be faceted. Scale bars: CLSM: 2 µm; cryo‐TEM: 500 nm (overview), 100 nm (inlet + facets).

The pepticombs were synthesized in two steps. First, the backbone consisting of 18 pentapeptide repeating units [(VPGKG)_18_] (hereafter K18) was recombinantly expressed in *E. coli* BLR (Figure 1A,B) as previously reported.^[^ ^31^ ^]^ K18 belongs to the family of elastin‐like supercharged unfolded polypeptides bearing one cationic lysine per pentapeptide.^[^ ^31^, ^32^ ^]^ We chose this peptide, as it provides a density of binding sites for hydrophobic tails similar to that at which iCPs were able to assemble into vesicles.^[^ ^26^ ^]^ The purity and molecular weight of K18 were qualitatively and quantitatively assessed by SDS page (Figure S1, Supporting Information) and MALDI‐ToF (Figure S2 and Table S1, Supporting Information) (*M*_w_  =  10187.496 g·mol^−1^). The supramolecular pepticombs were produced by an acid‐base reaction between the phosphoric acid of the hydrophobic DDP and the lysine's amines in the backbone. The formation of pepticombisome vesicles was carried out by thin‐film rehydration. Briefly, we deposited a 30 µl drop of a methanol/chloroform solution of pepticombs (c  =  10 mg·mL^−1^) onto Teflon plates and allowed the solution to dry. Subsequently, the plates were rehydrated in water overnight resulting in a suspension of vesicles (Figure 1D). We chose this method for its relative simplicity compared to alternatives like the injection method, electroformation, or emulsion transfer, which often involve the use of organic solvents that can persist in the membrane or sugars for hydration.^[^ ^33^ ^]^ Confocal and cryogenic transmission electron microscopy (cryo‐TEM) revealed the formation of predominantly unilamellar vesicles (**Figure** **2**A,B). We analyzed the distribution of diameters of 200 vesicles that resulted in a mean diameter of 3.31 µm with a standard deviation of 3.02 µm and a skewness of +2.27 (Figure S4, Supporting Information). The observed dispersity in diameter is typical for vesicles formed by thin film rehydration. The positive skewness suggests a right‐tailed distribution, as the majority of vesicles are <5 µm and only a few exceed 10 µm with the largest having a diameter of 17 µm. The most prominent vesicle morphology was spherical, while a fraction of vesicles displayed regions that were faceted (Figure 2C).

**Figure 3:**
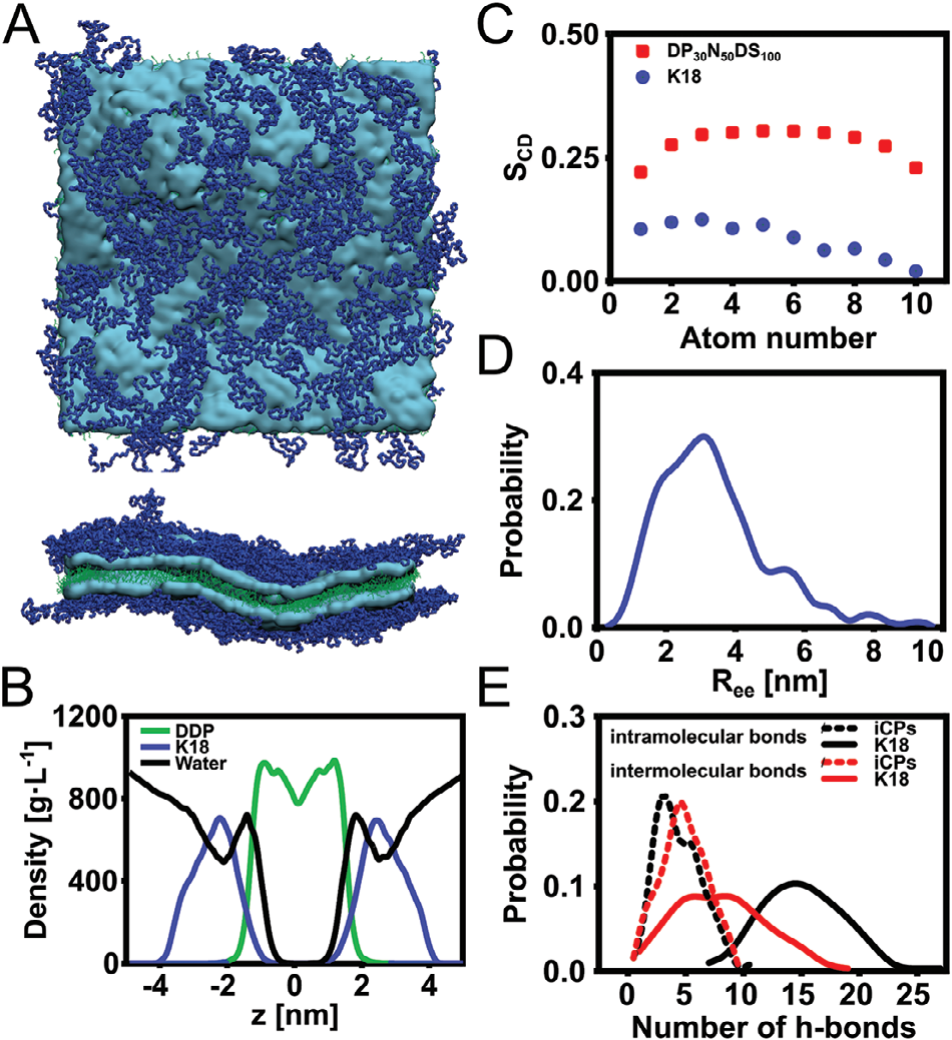
A) Snapshots of simulated bilayers of pepticombisomes. The top view image shows the randomly arranged peptide backbones (blue) on top of the DDP phosphate heads (cyan), and the side view (cross‐section along the normal of the membrane) displays the organization of DDP (tails = green, heads = cyan) and the coiling formation of the backbone. B) Density profile of DDP (green), peptide backbone (blue), and water (black) in pepticombisomes. C) Deuterium order parameter (S_CD_) of the DDP within the membrane of i‐combisomes (red) and pepticombisomes (blue). D) Distribution of the distances between K18 chain ends. The random distribution indicates random coil arrangement in contrast to the rod‐like arrangement of iCPs. E) A number of inter‐ and intramolecular hydrogen bonding of the synthetic backbones in iCPs (dashed line) and the K18 peptide backbone (solid line).

We conducted atomistic molecular dynamic simulations to gain deeper insight into the molecular arrangement within the membrane of pepticombisomes and their overall morphology. The simulations were performed by first allowing the assembly of DDP to which the peptide backbones were allowed to complex and equilibrate. The resulting bilayers displayed three distinct regions: a central DDP‐rich region flanked by a layer of SUP backbones on each side, as observable in the side‐view snapshots and density profile (**Figure** **3**A,B). The DDPs in the central region were organized with their tails inward, showing low penetration of water. This organization resembles a bilayer with a thickness of ≈4 nm, approximately double the thickness of a single DDP, indicating a very low if any, degree of interdigitation of the hydrophobic tails. Moreover, using an equilibrated state of the system we calculated the charge distribution across the membrane and the distance between the quaternary ammonium of protonated lysine residues and the phosphate anion of DDP using a radial distribution function (RDF). The former showed negligible charge at any position across the membrane (Figure S5A, Supporting Information). The RDF displayed a single peak at 3 Å followed by a monotonic increase afterward (Figure S5B, Supporting Information). The peak corresponds to the distance of the closest cation and anion. This short distance indicates that quaternary ammonium and the phosphate were tightly bound as if in a covalent bond. On the other hand, the lack of subsequent peaks in the RDF highlights that there were negligible interactions with the following neighbors further supporting the strength of the complexation. These results are in agreement with previous works on polyelectrolytes‐surfactant complexes.^[^ ^34^ ^]^ Consequently, the membrane of the pepticombisome has negligible surface charge as if it has been formed by the assembly of neutral amphiphiles.

Additionally, we calculated the deuterium order parameter (S_CD_), a metric that characterizes the orientational order of the alkyl chains. The S_CD_ displayed a maximum near the phosphate group and decreased monotonically, while constantly remaining lower than the S_CD_ for iCPs (Figure 3C; Figure S6, Supporting Information). This indicates a lower degree of packing of the hydrophobic tails in the pepticombisomes compared to the acrylamide‐based iCPs. This can be attributed to the lower density of cationic groups in the pepticombisomes (20% of amino acids appended with DDP), which enables freer movement of DDP within the hydrophobic domain. Consequently, this decreases intermolecular interactions between neighboring alkyl chains, lowers the packing efficiency, and results in lower S_CD_ values. Furthermore, the peptide backbone exhibits distinct differences from its synthetic counterpart. While in both systems, the backbones are restricted to quasi‐2D conformations, the iCPs's backbones acquired a rod conformation arranged in a nematic‐like fashion, a behavior not observed in the pepticombisomes. The persistence length for K18 in the membrane was ≈1.2 aminoacids (≈1% of K18 contour length) indicating that they were far from rod‐like behavior (Figure S7A, Supporting Information). The distribution of distances between K18 ends (R_ee_) has a mean and a standard deviation of the same order, indicating a quasi‐2D random coil (Figure 3D). Moreover, we additionally assessed the distribution of R_ee_ of peptides with half and double the length (K9 and K36), resulting in a scaling governed by the equation $R∼N^{3/5}$, which is commensurate with a coil with excluded volume interactions (Figure S7B,C, Supporting Information).^[^ ^35^ ^]^ The higher flexibility of the assembled pepticombs and their low degree of organization in the membrane, compared to the iCPs, is a consequence of the lower density of cationic groups (3–6 times) that are responsible for pinning the backbone to the interface.

As mentioned above, we observed giant and small unilamellar pepticombisomes with faceted regions (Figure 2C). There are many examples of faceted shells including viral capsids,^[^ ^36^ ^]^ halophilic archaea,^[^ ^37^ ^]^ and bacterial microcompartments.^[^ ^38^ ^]^ The elastic theory can explain the formation of icosahedral faceting in the one‐component system when the curvature energy concentrates on special areas (edges and vertices) that are separated by flat membranes. This is typically observed in liposomes assembled from lipids in a gel phase, i.e., long hydrophobic tails below their phase transition temperature. However, this does not explain the appearance of other polyhedral shapes as in this case. Moreover, the low S_CD_ values, like those observed for the pepticombisomes, and disordered backbones disfavor the necessary order to make the membrane rigid and faceted, suggesting a different mechanism for the facet morphology. Olvera de la Cruz demonstrated the formation of other Archimedean and Platonic polyhedral by highly charged bi‐component peptides or the inclusions of elastic heterogeneities.^[^ ^39^ ^]^

**Figure 4:**
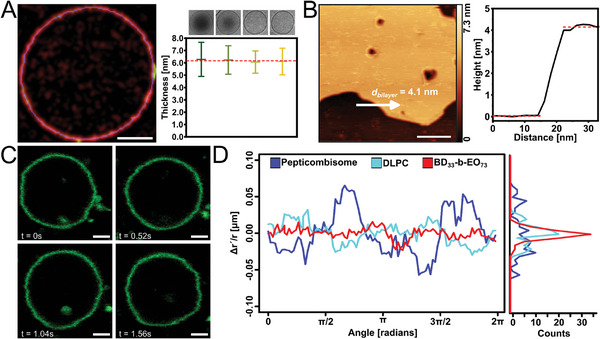
A) Skeletonization image of a cryo vesicle with the fitting line in the membrane and thickness plot showing the membrane thickness, the standard deviation, and the average thickness (red line) determined from four cryo‐TEM pepticombisomes. B) AFM topographic image of a pepticombisome bilayer on mica with the height plot determined along the line marked with an arrow. C) CLSM images of pepticombisome vesicles taken at a scan speed of 1000x with a resolution of 512 x 521 px showing the rapid fluctuations of the pepticombisome membranes. D) Angular fluctuations of radii (Δr´/r) after subtracting the first two harmonics of the cosine decomposition and distribution of fluctuations of pepticombisomes, DLPC liposomes, and BD33‐b‐EO73 block‐copolymers. Scale bars: AFM: 100 nm; CLSM: 2 µm.

The question remains, however, which intermolecular interaction is at play to stiffen the membrane of some vesicles? Percec et al. observed the formation of polygonal glycodendrimersomes, where the hydrophilic groups, mannose, could potentially form hydrogen bonds.^[^ ^40^ ^]^ Moreover, Zumbuehl demonstrated the formation of faceted D‐ and cubic‐shaped liposomes from 1,3 and 1,2‐diamidophospholipids via the formation of lateral hydrogen bonding, underscoring the importance of this subtle but cumulatively strong interaction.^[^ ^41^ ^]^ Thus, we analyzed the number of intra‐ and intermolecular hydrogen bonds in the pepticombs within the membrane. On average, each peptide formed 15.1 ± 3.5 intramolecular and 8.0 ± 3.9 intermolecular hydrogen bonds (Figure 3E, solid lines). In contrast, far fewer intermolecular hydrogen bonds were observed in iCPs (Figure 3E, dashed lines) and none for K18‐SDBS complexes.^[^ ^42^ ^]^ This explains why pepticombisomes display facets, while iCPs show hardly any. Moreover, the probability distribution of intermolecular H‐bonds for the pepticombs is skewed toward higher numbers, indicating that certain vesicles or regions of them could be enriched with hydrogen bonds, leading to localized facets. Several factors play an important role in mimicking biomembranes with artificial systems: a biomimetic membrane thickness, sufficient membrane flexibility to perform biological functions, and the ability to incorporate functional units into the membrane. First, we examined the thickness of the membrane of pepticombisomes as determined by simulations, by cryo‐TEM of vesicles, and by atomic force microscopy (AFM) of partly hydrated bilayers deposited on a flat substrate. The density profiles (Figure 3B) showed a bilayer thickness of ≈7 nm. The cryo‐TEM analysis of vesicles in their fully hydrated state yielded 6 ± 1 nm (**Figure** **4**A; Figure S8, Supporting Information), whereas AFM showed slightly lower values of 4.1 nm. This difference is caused by the partial collapse of the hydrophilic parts when the membranes are partly dehydrated (Figure 4B).

The flexibility of a membrane is inversely proportional to its bending rigidity (𝜅), which defines the energy required to deform a membrane and is directly related to the shape and stability of the vesicles. Controlling it allows mimicking biological functions such as division, fusion, endo‐, and exocytosis without the need for additional active components.^[^ ^43^ ^]^ Liposomes exhibit consistently fluctuating membranes, as their bending rigidity is just on the order of tens of the thermal energy (𝜅_DLPC_ ≈ 26 k_B_T).^[^ ^44^ ^]^ Likewise, pepticombisomes show strongly fluctuating membranes (Figure 4C; Video S1, Supporting Information). To investigate the fluctuations further, we analyzed the membrane contours of pepticombisomes and compared them with those of 1,2‐dilauroyl‐sn‐glycero‐3‐phosphocholine (DLPC) liposomes and polybutadiene‐*b*‐polyethylene oxide polymersomes (BD33‐b‐EO73) (Figure 4D; Figure S9, Supporting Information). The membrane fluctuations are visualized in terms of the relative angular distribution of radii ((Δr/r(𝜑)’) removing the influence of vesicle shape and size. Pepticombisomes displayed fluctuations similar to those of DLPC liposomes, although the probability function is much broader. In polymersomes, bending of the membrane requires the reorganization of entire macromolecules and thus depends on the molecular weight.^[^ ^17b^ ^]^ In pepticombisomes the ionic coupling allows freer movement of DDP between neighboring chains, facilitating reorganization, and thus decoupling molecular weight and flexibility. This and the overall low density of hydrophobic moieties result in vesicles even more flexible than liposomes.

**Figure 5:**
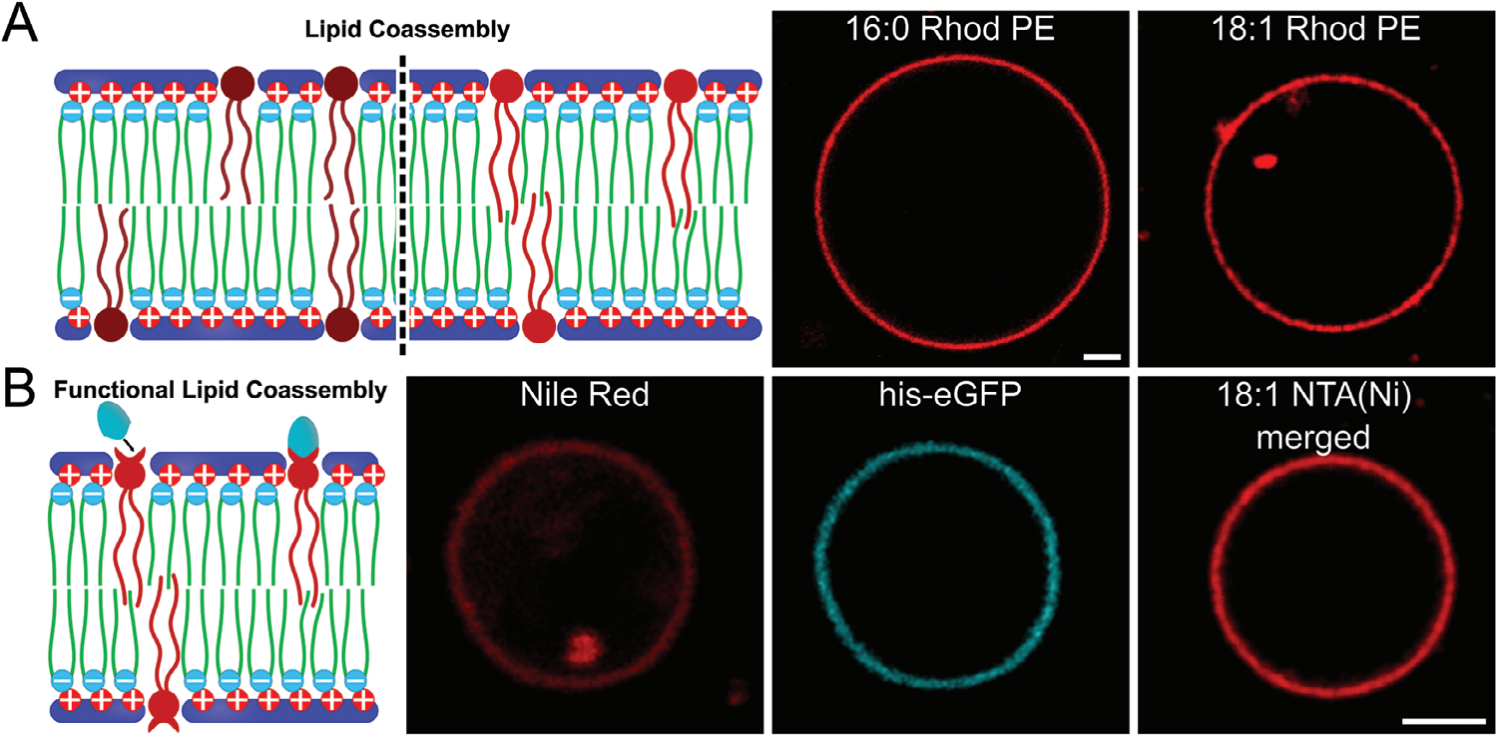
A) Coassembly of pepticombs with 0.1 mol% 16:0 Rhod PE and 1 mol% 18:1 Rhod PE. Left side: scheme depicting the introduction of each of the lipids. Right side: CLSM demonstrating the coassembly. Both lipids were uniformly distributed within the membrane. B) Functionalization of pepticombisomes using a 1 mol% 18:1 NTA(Ni) lipid and Nile Red for visualization of the membrane to which his‐tagged eGFP is complexed. Left side: scheme of the system. Right side: CSLM of a pepticombisome, displaying fluorescence from Nile Red, eGFP, and the merge. The channel of eGFP shows the uniform distribution of this protein across the whole surface. Scale bars: 2 µm.

The incorporation of functionality into membranes enables functions such as recognition, signaling, and the execution of reactions. A promising strategy is the design of heterogeneous systems by the integration of functional amphiphiles. This can be achieved by co‐assembly of membrane‐forming molecules with different heads than those of the pepticombs. However, a membrane with biomimetic thickness without hydrophobic mismatch is a fundamental prerequisite to enable integration.^[^ ^18f^ ^]^ Incorporation of functional moieties has already been observed in various vesicle systems. Apart from serving a structural purpose like cholesterol,^[^ ^45^ ^]^ synthetic vesicles have been functionalized with glycans,^[^ ^23f^ ^]^ DNA,^[^ ^46^ ^]^ DNA pores,^[^ ^47^ ^]^ proteins,^[^ ^48^ ^]^ as well as lipids serving as environmental sensors^[^ ^49^ ^]^ or chemo‐ and photoresponsive switches.^[^ ^50^ ^]^ But is homogeneous functionalization also possible with pepticombisomes? To answer this question, we investigated the possibility of integrating lipids into our membrane (Figure 1E). We formed hybrid vesicles by co‐assembling pepticombs with the rhodamine‐labeled phospholipids 16:0 Rhod PE and 18:1 Rhod PE at 0.1 and 1 mol%, respectively (**Figure** **5**A). Confocal images show a uniform distribution of dye across the membrane indicating a homogeneous mixture of lipids and pepticombs attributed to excellent hydrophobic matching of the components, regardless of the length of the lipid's hydrophobic tails. We then performed a co‐assembly with 1 mol% of 18:1 NTA(Ni) lipid to introduce a functional handle into the membrane where other molecules can be attached (Figure 5B). The subsequent addition of his‐tagged eGFP (Figure S3 and Table S2, Supporting Information) resulted in immediate binding across the membrane, demonstrating a uniform distribution of the lipid while maintaining its functionality. Mixing pure pepticombisomes labeled with Nile Red with his‐eGFP on the other hand did not result in any visible accumulation of fluorescent protein on the vesicle periphery after over 1 h (Figure S10, Supporting Information).

## 3 Conclusion

In summary, this work demonstrates unprecedented peptide combs that are used for the formation of giant unilamellar vesicles. With a combination of biophysical techniques and simulations, we explore the molecular arrangement and properties of our pepticombisome membranes. In contrast to their synthetic counterpart, iCPs, pepticombs hold the striking feature of a sequence‐defined backbone resulting in a homogeneous distribution of ligands and ensuring identical topology. Interestingly, we discovered that forcing the backbone into a 2D conformation at the interface combined with the capability of the recombinant peptide backbone to exert intermolecular hydrogen bonding enables to generate elastic heterogeneities as well as a faceting transition so far not observed in a purely monocomponent and sequence‐defined macro/supramolecular system.

Remarkably, this did not require to have the hydrophobic tails in a gel phase. On the contrary, the free movement of DDP in the hydrophobic domain and the ionic linkage to the polypeptide backbone allow for remarkably flexible membranes, while retaining a biomimetic bilayer thickness. Furthermore, we successfully incorporated functional moieties, underscoring the biomimetic nature of pepticombisome membranes. Looking ahead, the versatility of pepticombs offers a promising avenue for further exploration. The system adaptability in membrane building blocks allows for tunable parameters such as peptide sequence, length, degree of substitution, and alkyl tail length. This flexibility opens ways for developing pepticomb systems that facilitate external function incorporation, vesicle fusion, and potentially artificial phagocytosis. Moreover, the use of recombinant polypeptides provides the opportunity to integrate desired functionalities and receptors directly into the building blocks of the vesicle membrane, even enabling the hybridization with other information‐rich biomolecules such as DNA or RNA, obviating the integration of functional amphiphiles. Thus, this work lays the foundation for future exploration and development in the realm of biomimetic peptide comb polymer vesicle technology.

## Conflict of Interest

The authors declare no conflict of interest.

## Supporting information



Supporting Information

Supplemental Video 1

## Data Availability

The data that support the findings of this study are available from the corresponding author upon reasonable request.
