# Supplementary material for: Pepticombisomes: Biomimetic Vesicles Crafted From Recombinant Supercharged Polypeptides with Uniformly Distributed Side‐Chains
Source: Adv Sci (Weinh). 2025 Feb 22;12(15):2411497. doi: 10.1002/advs.202411497 (PMC12005736; doi:10.1002/advs.202411497)
Supplement: Supplementary file 1 — Supporting Information [file ADVS-12-2411497-s001.pdf]

ADVANCED SCIENCE
Open Access

Supporting Information

for *Adv. Sci.*, DOI 10.1002/advs.202411497

Pepticombisomes: Biomimetic Vesicles Crafted From Recombinant Supercharged Polypeptides with Uniformly Distributed Side-Chains

Dominik Söder, Melina Schadt, Vladislav S. Petrovskii, Tamás Haraszti, Khosrow Rahimi, Igor I. Potemkin, Nina Yu. Kostina, Cesar Rodriguez-Emmenegger and Andreas Herrmann**

Supporting Information

Pepticombisomes: Biomimetic Vesicles Crafted from Recombinant Supercharged Polypeptides with Uniformly Distributed Side-Chains

Dominik Söder[a,b]†, Melina Schadt[a,b]†, Vladislav S. Petrovskii[c], Tamás Haraszti[b], Khosrow Rahimi[b], Igor I. Potemkin[b,c], Nina Yu. Kostina[d], Cesar Rodriguez-Emmenegger*[b,d,e,f], Andreas Herrmann*[a,b]

[a] D. Söder, M. Schadt, Prof. A. Herrmann
Institute of Technical and Macromolecular Chemistry
RWTH Aachen University

[b] D. Söder, M. Schadt, Dr. T. Haraszti, Dr. K. Rahimi, Prof. I. I. Potemkin, Prof. C. Rodriguez-Emmenegger, Prof. A. Herrmann
DWI – Leibniz Institute for Interactive Materials
Forckenbeckstraße 50, 52074 Aachen, Germany
Worringerweg 2, 52074 Aachen, Germany

[c] V. S. Petrovskii, Prof. I. I. Potemkin
Physics Department
Lomonosov Moscow State University
Leninskie Gory 1-2, 119991 Moscow, Russian Federation

[d] Dr. N. Yu. Kostina , Prof. C. Rodriguez-Emmenegger
Institute for Bioengineering of Catalonia (IBEC)
Carrer de Baldiri Reixac, 10, 12, 08028 Barcelona, Spain.

[e] Prof. C. Rodriguez-Emmenegger
Institució Catalana de Recerca i Estudis Avançats (ICREA)
Passeig Lluís Companys 23, 08010 Barcelona, Spain

[f] Prof. C. Rodriguez-Emmenegger
Biomedical Research Networking, Center in Bioengineering, Biomaterials and Nanomedicine,
The Institute of Health Carlos III, Av.Monforte deLemos 3–5, 28029, Madrid, Spain

E-mail: herrmann@dwi.rwth-aachen.de, rodriguez@dwi.rwth-aachen.de

† These authors contributed equally to this work.

1. Materials and Methods

Materials, Reagents, and Biochemicals

Anhydrous (over molecular sieves) methanol (MeOH, 99.8%) and chloroform ($CHCl_3$, 99,9%) were obtained from Acros Organics. The membrane dyes Bodipy FL and Nile Red were obtained from Sigma Aldrich and the ligand didodecyl hydrogen phosphate (DDP) from abcr. 1,2-dilauroyl-sn-glycero-3-phosphocholine (12:0 PC, DLPC), 1,2-(dipalmitoyl or dioleoyl)-sn-glycero-3-phosphoethanolamine-N-(lissamine rhodamine B sulfonyl) (ammonium salt) (16:0 or 18:1 Rhod PE), and 1,2-dioleoyl-sn-glycero-3-[(N-(5-amino-1-carboxypentyl)iminodiacetic acid)succinyl] (nickel salt) (18:1 NTA(Ni)) were purchased from Avanti Lipids. The BD_{33}-b-EO_{73} block-copolymer (BCP) was obtained from Polymer Source, Inc. Secure Seal spacers were bought from Thermo Fisher Scientific and the OTESPA-R3 AFM tips from Bruker. The ultra pure water used in this research (18.2 MΩ cm at 25 °C) was obtained from PURELAB flex (Veolia water, France). Biochemicals for SUP expression were purchased from different suppliers. LB media and LB agar as well as isopropyl *β*-D-1 thiogalactopyranoside were purchased from Sigma Aldrich, components for expression media like yeast extract and tryptone were purchased from BD (France), glycine from Acros organics (Belgium), carbencilin for antibiotical selection was purchased from Duchefa Biochemistry (Netherlands) and potassium dihydrogen phosphate and dipotassium hydrogen phosphate from abcr. Chemicals for protein expression like magnesium chloride, sodium dihydrogen phosphate, sodium chloride BioUltra ≥ 99.5%, and imidazole puriss p.a. ≥ 99.5 % were purchased from Sigma Aldrich, disodium hydrogen phosphate from Alfa Aesar, DNase I and cOmplete^{TM}, EDTA free from Roche (Switzerland). For protein characterization 1.4-dithio-DL-threitol, acrylamide/bis-acrylamide solution (37.5: 1), and ammonium persulfate were purchased from Carl Roth, bromophenol blue from Alfa Aesar, Coomassie Brilliant blue, *N*,*N*,*N'*,*N'*-tetramethylethylenediamine from Sigma Aldrich and PageRuler^{TM} Prestained Protein Ladder from Thermo Scientific (UK). Super-DHB (Santa Cruz Biotechnology) was used as matrix for MALDI-TOF. The pET25b expression vector was purchased from Thermo Scientific (Germany). Expression host *E. coli* BLR (DE3) was purchased from Merck (Germany). All materials and reagents were used as received unless stated otherwise.

Methods

Preparation of recombinantly expressed proteins

Protein expression: The genetic information of K18 was cloned into pET25b(+) and was transformed in chemo competent *Escherichia coli* BLR (DE3). 1000 mL Terrific Broth (12 g·L^{-1} tryptone, 24 g·L^{-1} yeast extract, 2.2 g·L^{-1} KH_2PO_4, 9.4 g·L^{-1} K_2HPO_4, 4 mL·L^{-1} Glycerol with 100 µg·mL^{-1} Carbencillin) was inoculated with an overnight culture and incubated at 37°C until an OD_{600} 0.6- 0.8 was reached. Then, the culture was induced with 1 mM IPTG and incubated for 22 h at 30°C. For the cell harvesting, the expression culture was centrifuged at 8.000 x *g* for 20 min. The pellet was solved in 2.5 mL IMAC buffer A (300 mM NaCl, 50 mM $NaPO_4$, 20 mM Imidazole, pH 7-8) per g pellet and proteinase inhibitors, DNase I and $MgCl_2$ were added. Afterwards the cells were lysed via high pressure (20 kpsi) and then incubated at room temperature for 2 h. The cell debris was removed via centrifugation for 30 min at 22.000 x *g*.

The supernatant was filtered through 0.2 µm filters. His-tagged eGFP used for the binding to NTA(Ni) lipids was expressed via the same procedure.

Protein purification: The protein was purified in a two-step process. The ion metal affinity chromatography (IMAC) using a Ni-NTA column (HisTrap FF, Cytiva) was followed by a cation exchange chromatography (Hi Trap Heparin, Cytiva). The chromatographies were performed according to manufacturer's instructions using a gradient elution from 0 % to 100 %, respectively. The protein was desalted using the PD-10 desalting columns (GE Healthcare) after manufacturers instruction and was afterwards lyophilized.

Characterization of SUPs

Sodium didodecyl sulfate polyacrylamide gel electrophoresis (SDS-PAGE): The purified proteins were analyzed by SDS-PAGE with a 12% polyacrylamide and stained with Coomassie Solution (40% ethanol, 10% glacial acetic acid, 1 $g \cdot L^{-1}$ Brilliant Blue R250). The gels were visualized using the ChemiDoc Imaging System (BioRad).

Matrix-associated laser desorption/ionization - time of flight mass spectroscopy (MALDI-TOF): MALDI-TOF was used to further analyze the purity of K18. The salt-free protein sample was blended 1:1 with Super-DHB (50 $mg \cdot mL^{-1}$ in 50:50 (v/v) Acetonitrile-TFA). The expected mass of K18 was 10224.11 Da.

Synthesis of ionically-linked pepticombisomes

The positively charged SUPs were complexed with DDP to form-pepticombs. For this, MeOH was added to the lyophilized SUPs to obtain a concentration of 1 $mg \cdot mL^{-1}$. The solution was sonicated until the SUPs were homogeneously distributed. Afterwards, the solution was centrifuged (12,000 rpm, 2 min, RT) to remove undissolved SUP. The supernatant was dried and dissolved again in MeOH. For the complexation, DDP in $CHCl_3$ (c = 40 $mg \cdot mL^{-1}$) was added dropwise to a stirring solution of SUPs in MeOH at RT in the amount necessary to obtain quantitative complexation. The concentration of the SUP solution was adjusted before addition of DDP to obtain a final complex concentration of 10 $mg \cdot mL^{-1}$. After stirring for 2 h the complex was stored at 4°C.

Vesicle formation

Thin film rehydration method: This method was performed to obtain µm-sized pepticombisomes. 30 µl of amphiphiles (c = 10 $mg \cdot mL^{-1}$) in MeOH:$CHCl_3$ were mixed with 1 mol% Bodipy or 0.1 mol% Nile Red and deposited on a roughened Teflon plate. After drying at RT, followed by drying in vacuum for 2 h, 300 µl Milli-Q water were added onto the plate to rehydrate the film at 60°C overnight yielding a final concentration of 1 $mg \cdot mL^{-1}$. DLPC (c = 10 $mg \cdot mL^{-1}$ in $CHCl_3$) vesicles were formed identically. BD_{33}-b-EO_{73} (c = 10 $mg \cdot mL^{-1}$ in $CHCl_3$) vesicles were rehydrated at 80°C.

Vesicle characterization and analysis of membrane properties

Confocal laser scanning microscopy (CLSM): CLSM was performed to observe the formation, size, and morphology of pepticombisomes formed by thin film rehydration, the membrane fluctuation, as well as the co-assembly with lipids. Measurements were performed on a Leica TCS SP8 confocal microscope (Wetzlar, Germany) with a 63x/1.40 glycerol-immersion objective and PMT or HyD detectors. The temperature during the measurements was kept constant at 22°C. Two high precision cover glasses (170 ± 5 µm) with 20 µl of pepticombisome

solution ($c = 1$ mg·mL^{-1}) in between were sealed with SecureSeal™ spacers (d = 13 mm, h = 0.12 mm). Image cropping and contrast/brightness correction were done using the Fiji-ImageJ software.[1] Vesicle diameters were manually measured in the Leica LASX software.

Cryogenic transmission electron microscopy (cryo-TEM): cryo-TEM was utilized to observe the morphology of pepticombisomes and to determine the bilayer thickness. The measurements were performed with a Zeiss LibraTM 120 TEM (Oberkochen, Germany) at -168°C with an applied electron beam acceleration voltage of 120 kV. Sample preparation was performed with a FEI Vitrobot (Model Mark IV) plunge freezing station by deposition of an aqueous pepticombisome solution (5 µl, 1 mg·mL^{-1}) on plasma-treated lacey grids. Samples were blotted and shock frozen in liquid ethane, before being fixed on Model 910 cryo transfer specimen holder from Gatan (Pleasanton, USA). Images were taken with an in-column Omega energy filter with CCD detector.

Atomic force microscopy (AFM): A diluted dispersion of pepticombisomes prepared by thin-film rehydration in water ($c = 0.3$ mg·mL^{-1}) was drop casted on freshly peeled Mica. The film was dried at RT for 2 h before measurements. Images were recorded as topological scans in tapping mode (Multimode Atomic Force Microscope NanoScope V (Digital Instruments)) using OTESPA-R3 silicon probes with a nominal spring constant of 26 N·m^{-1} and a tip radius of 7 nm. The images were analyzed using the Gwyddion software.[2]

Atomistic molecular dynamics simulation: Molecular dynamics were carried out with the Gromacs 2020.6 package with a constant temperature of 300 K.[3] We used the NPT ensemble at a pressure of 1 atm to obtain a local thermodynamic equilibrium before continuing the simulation with the NVT ensemble. In both ensembles, the temperature was coupled to a velocity-rescale thermostat,[4] while pressure coupling was set by the Berendsen barostat. Periodic boundary conditions were applied in all directions and the motion equations were integrated with a time step of 2 fs. The cut-off length of the Lennard-Jones potential was 1.2 nm. The long-range electrostatic interaction was calculated using the PME method.[5] The LINCS algorithm constrained bond vibrations.[6] The OPLS-AA force field[7] describes the interaction in phospholipid molecules and polymers with SPC/E[8] model for water. Atomistic dynamic simulations were performed to provide insight into the molecular arrangement in the pepticombisome membrane according to the protocol mentioned above. To obtain a bilayer, we arranged 2688 surfactant molecules in hexagonal close-packed order in the midplane of a 31.50 x 31.25 x 17.12 nm^3 sized box. The number of surfactant molecules remained constant in all simulated systems. First, sodium atoms were added to the bilayer to neutralize the charge of the system, followed by equilibration of the bilayer, which was well equilibrated after 100 ns. Then the z-direction of the box was extended to 20 nm and 298, 150, and 74 peptide molecules were added for K9, K18, and K36, respectively. The peptide-backbones were considered monodisperse in the simulation. Therefore, the peptide-backbones were placed in equal quantities on both sides of the bilayer. The further equilibration was carried out with two stages with NVT and then NPT ensamles. The last 10 ns of the simulated trajectories were used to analyze the data. Figure 3A in the manuscript shows the final state of the simulated K18 pepticombisome membrane. The membrane consists of three zones containing a hydrophobic core domain of DDP flanked by the hydrophilic peptide-backbone. The snapshots revealed no visible order of backbone in the membrane. To calculate a density profile (Figure 3C) the xy-plane was divided into small squares with the z-plane set as the normal line. The defined squares contained a flat section of the bilayer. From each square, the respective density profile was calculated and then averaged over all squares. Also, the density profile was used to calculate the thickness of a pepticombisome membrane. We chose the distance on the density

plot between upper and bottom leaflets where 90% of the mass is located. The snapshots showed that the peptides acquired no distinguishable preferential organization or order in the bilayer, but rather form loops and expand from the surface. This suggests packing of the DDP ligands, leading to loops of non-complexed amino acids. The order in the bilayer was further quantified by the deuterium order parameter S_{CD} according to the following equation: $S_{CD} = \langle (3cos^2\theta - 1)/2 \rangle$ where theta is the angle between bilayer normal and the vector lined from carbon atom to hydrogen atom in an alkyl tail of phospholipid. Figure S6 shows the carbon atoms that were used for the calculation. The second tail was numbered similar to the first tail.

The last 20 ns of the simulation trajectory were used to calculate the average charge along the z-axis of the simulation box (Figure S5A). The box was divided into 100 slices with a step size of 0.17 nm, and the charge located in each slice was averaged along the x- and y-axes.

The radial distribution function (RDF) shows how many particles are located at a distance *r* from a particle within a shell of width *dr*. In this study, RDF was used to describe the mutual distribution of charged groups in the membrane (Figure S5B).

The persistence length was calculated from the system trajectory using a definition: $\langle cos\varphi \rangle = e^{-L/P}$, where L is the distance along the chain at which correlations decrease by a factor of *e*. To study the behavior of the peptide on the membrane surface, an analysis of the distances between the peptide chain ends (end-to-end distance or R_{ee}) was performed using the last 20 ns of the trajectory (Figure S7B).

Membrane fluctuation analysis: We investigated the undulations of pepticombisome, DLPC liposome, and BD_{33}-b-EO_{73} BCP membranes by CLSM. Vesicles were prepared by thin film rehydration with 1 mol% Bodipy as described above. 16 µl of vesicles were mixed with 4 µl HEPES (10 mM) to deflate the vesicles. Images were taken at a scan speed of 1000 Hz with a resolution of 512 x 512 pixels. A detailed analysis of the contours can be found in the Supporting Results section.

Vesicle functionalization

Co-assembly of pepticombs with lipids: To determine whether pepticombs mix homogeneously with lipids, we prepared hybrid vesicles by thin film rehydration. Pepticombs were co-assembled with 16:0 Rhod PE lipid (0.1 mol%), 18:1 Rhod PE lipid (1 mol%), and 18:1 NTA(Ni) lipid (1 mol%). 0.1 mol% Nile Red dye were added to the latter for visualization. All samples were observed in CLSM. To determine the binding of his-eGFP to the NTA(Ni) lipid 1 µl of his-eGFP (c = 1 mg·mL^{-1} in water) was added to 19 µl of vesicles.

2. Supplementary Results

2.1 Protein expression and characterization

Expression of SUPs

The gene sequence of the plasmid carrying the information of K18 (pET25b-K18) was verified via Sanger Sequencing. The K18 peptide consists of 112 amino acids, containing 18 pentapeptide repeating units (VPGKG) with 18 positively charged lysine residues, as well as a 6x-poly-histidine-Tag at the C-terminus (Table S1).

Gene sequence K18:

atggctggccaaggtgtgggtgtaccaggaaaaggtgtaccagggaaaggagtaccaggaaaggggtacctggtaaagga
gttccaggtaagggagttccgggtaaaggagtgccggggaagggtgttccaggtaaaggtgttcctggaaaagtgccaggtgtg
ggtgtaccaggaaaaggtgtaccagggaaaggagtaccaggaaaggggtacctggtaaaggagttccaggtaagggagtt
ccgggtaaaggagtgccggggaagggtgttccaggtaaaggtgttcctggaaaagtgccaggtgggccctggcatcatcacca
tcaccactg

Protein sequence K18:

MAGQGVGVPGKGVPGKGVPGKGVPGKGVPGKGVPGKGVPGKGVPGKGVPGKVPGVGV
PGKGVPGKGVPGKGVPGKGVPGKGVPGKGVPGKGVPGKGVPGKVPGGPWHHHHHH

Table S1. Molecular weight, charge, and extinction coefficient of K18.

**Molecular weight (calculated)**	10224.110 Da
**Molecular weight (measured)**	10187.496 Da
**Net charge**	+ 18
**Extinction coefficients**	5500

The protein purity is analyzed using SDS-Page (Figure S1). The ~ 10 kDa protein runs slightly above the 10 kDa standard and was thus verified by MALDI-TOF. The calculated mass of K18 is 10224.11 Da, close to the confirmed mass via MALDI-TOF of 10187.496 Da (Figure S2). The lysine residues give the protein a net charge of +18 under physiological conditions. K18 has an extinction coefficient of 5500 (Table S1).

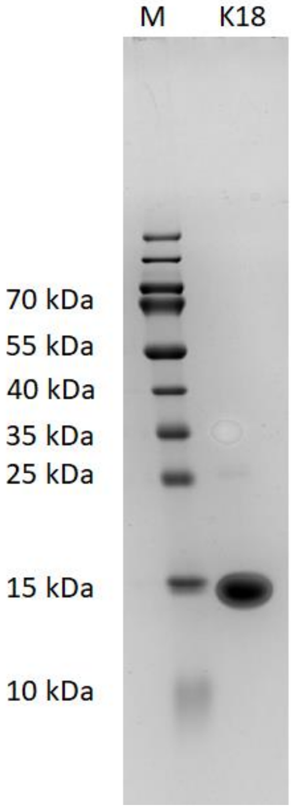

Figure S1. Approximate determination of the molecular weight of K18 using SDS-PAGE. The expressed and purified proteins with a molecular weight of 10.18 kDa run between the 15 kDa and the 10 kDa bands of the standard (M).

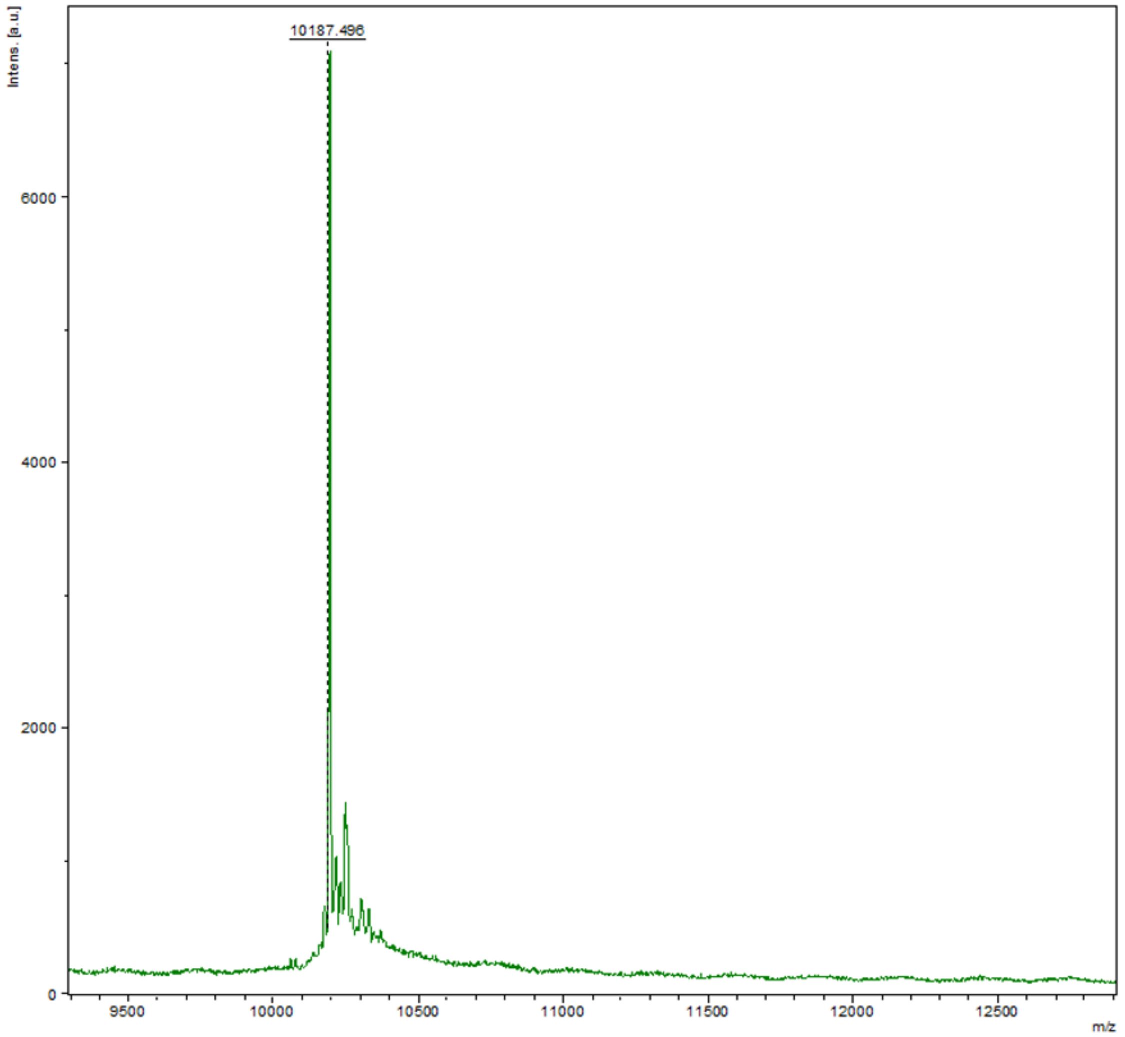

m/z	S/N	Quality Fac.	Res.	Intens.	Area
5088.21	21	965	4676	567	1451
10187.5	173	22799	6093	461	825
10252.43	9	32.8	6534	407	68.1

Figure S2. MALDI-TOF measurement of K18. The measured molecular weight of 10187.496 Da differs slightly from the calculated mass of 10224.11 Da.

Expression of eGFP

His-eGFP was expressed to test the binding to the NTA(Ni) functionalized lipid. The gene and protein sequences, the projected weight (Table S2) and the purity assessment by SDS-PAGE are shown below (Figure S3).

Gene sequence his-eGFP:

atggctggccaaggtgtgggtggcagcggtggctccgatagcacggagagtcttttcacaggggtcgtgccaattcttgtcgaactt
gatggcgacgtaaatggtcacaagttctcagtgcgcggggagggagaaggagatgccacaaacgggaaactgacgctgaaa
ttcatttgcaccaccggaaaattgccagtaccttggccgacactggtaacgacgttaacctatggagtgcaatgcttttcacgctatc
ctgaccacatgaaacagcatgattttttaagtctgctatgccagaaggctatgtgcaggaacgcactattacattcaaagatgacg
gaacatataagactcgcgcggaggtcaaattcgagggtgacaccttggttaaccgtatcgaactgaagggtattgatttcaaaga
agacgggaacattcttgggcataagctggaatacaattacaattcgcataatgtctatattacggcagacaaacaaaaaaacgg
catcaaagcaaatttcaaaatccgccacaatgtcgaagacggctcggtgcaacttgcagaccactaccaacagaatacaccga
ttggggacggtccagttctgttgccagacaaccactatttgtccactcagtccaaattatcgaaagaccccaatgagaagcgtgac
cacatggtactgctggagtttgtaaccgccgctgggattactggaggttcaggcggttcgccaggtgggcatcatcaccatcacca
ctg

Protein sequence his-eGFP:

MAGQGVGGSGGSDSTESLFTGVVPILVELDGDVNGHKFSVRGEGEGDATNGKLTLKFICTT
GKLPVPWPTLVTTLTYGVQCFSRYPDHMKQHDFFKSAMPEGYVQERTITFKDDGTYKTRAE
VKFEGDTLVNRIELKGIDFKEDGNILGHKLEYNYNSHNVYITADKQKNGIKANFKIRHNVEDG
SVQLADHYQQNTPIGDGPVLLPDNHYLSTQSKLSKDPNEKRDHMVLLEFVTAAGITGGSGG
SPGGHHHHH

Table S2. Molecular weight and extinction coefficients of his-eGFP.

**Molecular weight (calculated)**	28078.300 Da
**Molecular weight (measured)**	29489.096 Da
**Extinction coefficients**	19035

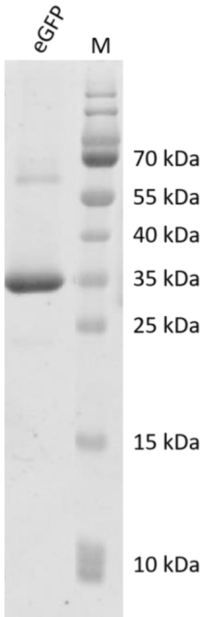

Figure S3. Approximate determination of the molecular weight of his-eGFP using SDS-PAGE. The expressed and purified proteins with a molecular weight of 29.49 kDa run below the 35 kDa band of the standard (M).

2.2 Determination of vesicle size and membrane properties

Determination of vesicle size

Thin film rehydration typically yields a polydisperse vesicle population. To obtain an average 200 vesicles diameters were manually measured. The data was plotted and analyzed to obtain the average diameter, the standard deviation, as well as the skewness of the data curve (Figure S4).

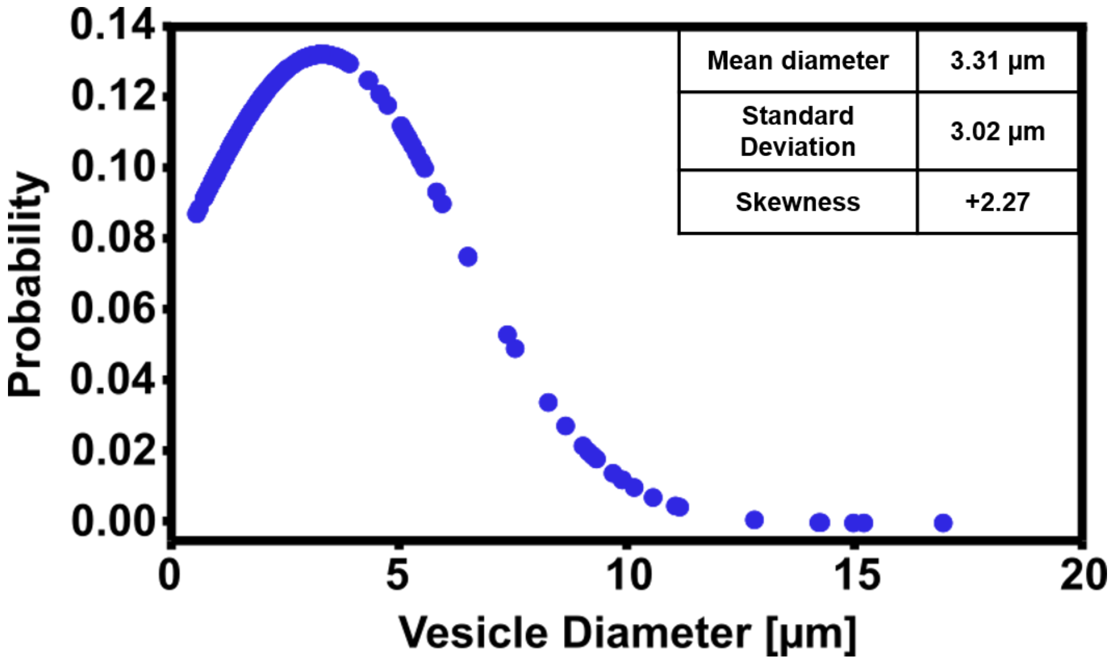

Figure S4. Distribution of diameters of 200 pepticombisomes.

Surface charge

Pepticombs form through the charge driven complexation between anionic DDP and cationic lysine residues in the peptide backbone. To investigate the effect of those charges on the overall membrane charge we calculated the charge across the membrane of a pepticombisome (Figure 5A). Furthermore, we calculated the distance between the anions and cations in the bonds using a radial distribution function (RDF) (Figure S5B).

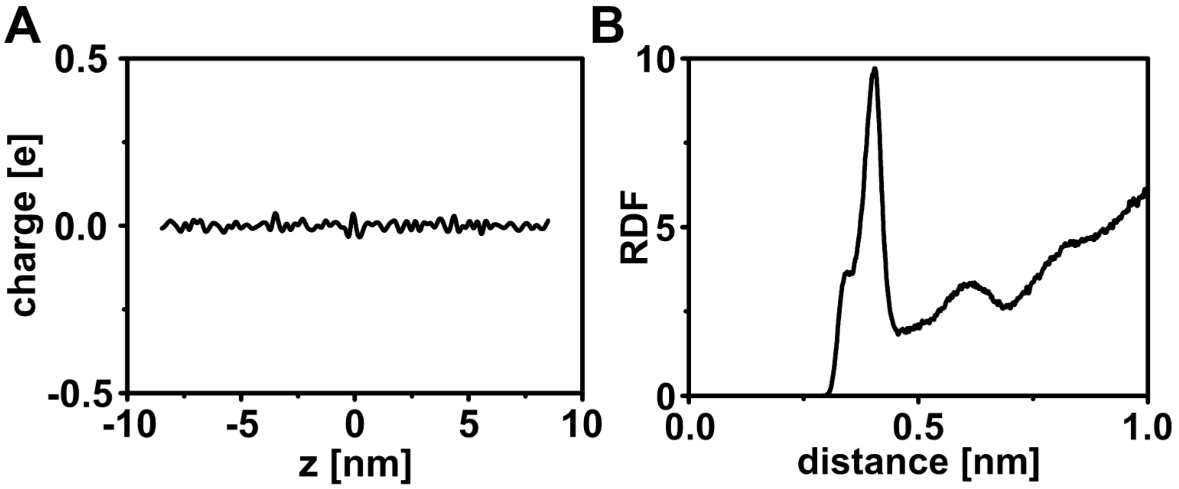

Figure S5. (A) Charge distribution across a pepticombisome membrane in equilibrated state. The membrane was divided in 100 slices of 0.17 nm and the charge was in each slice was averaged along the axes. (B) Distance between the phosphate anion of DDP and the quaternary ammonium of protonated lysine obtained using a radial distribution function.

Atomistic molecular dynamic simulations

The self-assembly of pepticombs cannot be accurately represented with geometrical models due to their complexity and size. To gain insight into the structure of pepticombisome membranes we performed atomistic molecular dynamic simulations as described above. The order in the bilayer was quantified by the deuterium order parameter S_{CD}. Figure S6 shows the carbon atoms that were used for the calculation. Both tails were numbered equally.

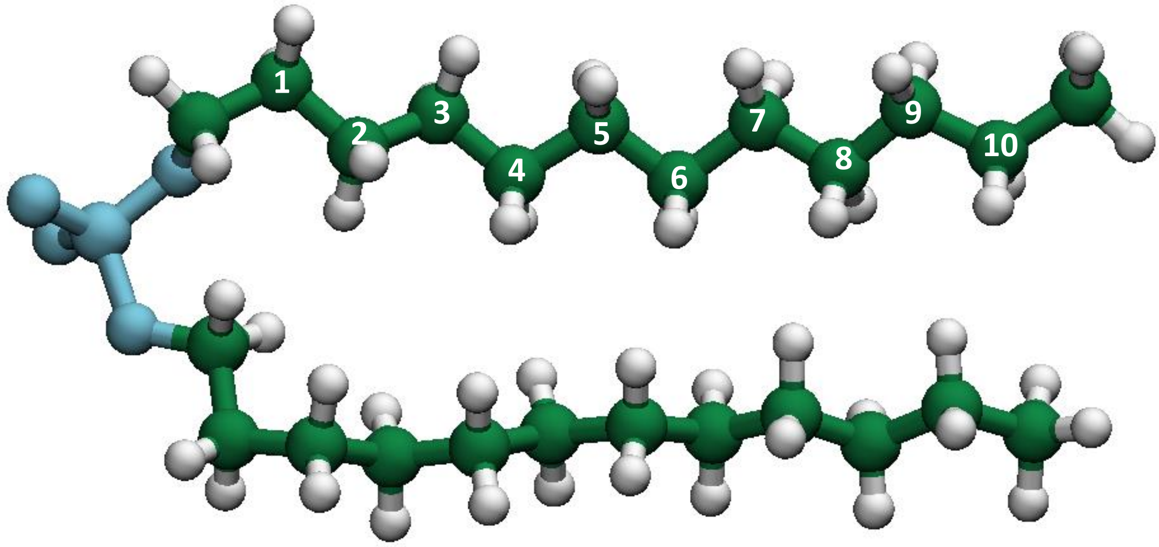

Figure S6. Atomistic representation of DDP used for the molecular atomistic simulations. The white numbers denote the atom numbers for the calculation of S_{CD} (Figure 3D, Manuscript).

The backbones of iCPs acquire a rod-conformation which arranged in a nematic-like fashion. To verify the order of pepticombs in the membrane we analyzed the persistence length for K18 in the membrane (Figure S7A). We also obtained the distribution of distances between K18 ends (R_{ee}) and compared them to peptides with half and double length (K9 and K36) (Figure S7B, C).

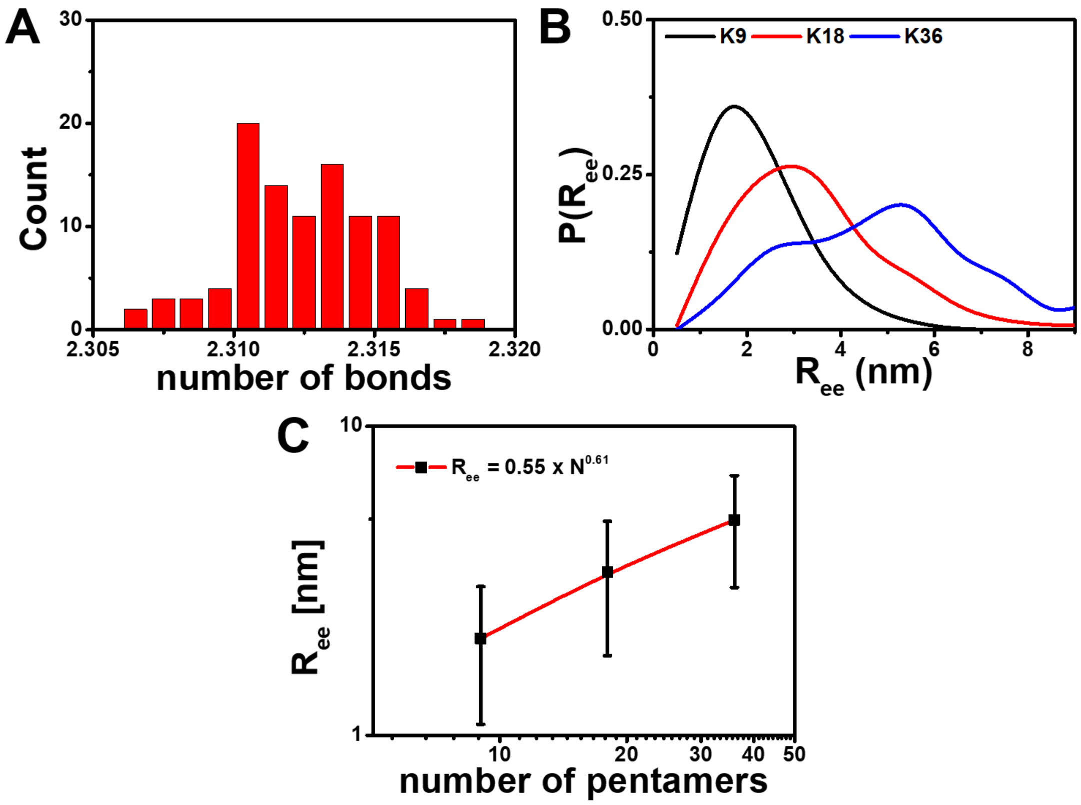

Figure S7. (A) Distribution of “persistent length” of K18, (B) Probability distribution of end-to-end distance for pepticombisomes formed from K9, K18, and K36 peptides, (C) Fitting of the average end-to-end distance by the power law.

Determination of the membrane thickness of pepticombisomes by cryo-TEM

The pixels of the cryo-TEM images were converted using a distance filter. Afterwards the central positions within the membrane were selected by a skeletonization (Figure S8). The distance values of the central positions to the membrane edge provided a distribution of half thicknesses.

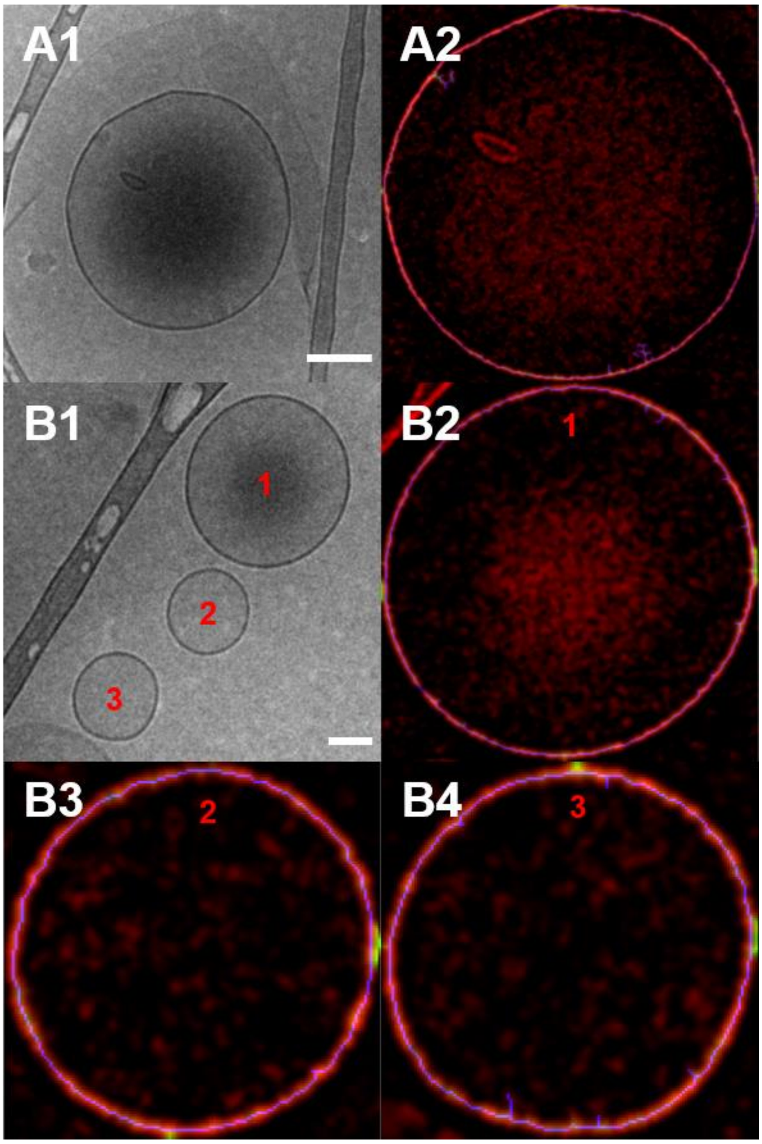

Figure S8. Determination of the bilayer thickness by cryo-TEM. (A1, B1) Cryo-TEM images and (A2, B2, B3, B4) the corresponding converted images including the fitting lines (blue) within the membrane obtained by skeletonization. Scale bars: 100 nm.

Analysis of membrane fluctuations

The molecular arrangement in membranes determines their ability to fluctuate. To compare the fluctuations of vesicles qualitatively, pepticombisomes, liposomes (DLPC), and polymersomes (BD_{33}-b-EO_{73}) were deflated. CLSM imaging showed constantly undulating membranes after deflation. To assess the fluctuations, single CLSM images of unilamellar vesicles of similar size were processed using a custom-made python script, based on a general procedure (Figure S9).[9] Objects around the studied vesicles were removed before processing

using the Fiji ImageJ software.[1] A background image was created for each sample by convolution of a Gaussian kernel (window size of 61 pixels, width σ = 10 pixels) and subsequently subtracted from the original image. The obtained negative pixels were set to zero. A second Gaussian kernel (window size of 21 pixels, width σ = 3 pixels) was applied to smoothen the image. A binary image was created from the results by applying a threshold calculated based on Otsu´s method.[10] Confluent images with a predefined size range were selected from the binary images. The size range was typically ranging from 500–15000 pixels area for each image, where the center of the mass defined the center of the object. The pixel coordinates were transformed to polar coordinates and angles relative to the center of the object. An angle range was defined from 0 to 2π rad with 100 data points. In each of the defined angle bins, the maximum was identified, then a local straight line was fitted in both directions from the maximum and the crossing point used as the corresponding radius.[11] The exported profiles were further processed using R. The mean radius was subtracted from each data set ($\Delta r = r - \langle r \rangle$), which allowed to compare the angular profiles. By shifting of $\Delta r(\varphi)$ the zero angle was set to the maximum of the curve (main axis of a prolate) for each vesicle. Afterwards $\Delta r(\varphi)$ was fitted with a series of cosine functions with integer harmonics where φ is the angle obtained from the data set: Δr = $\sum \alpha i \cdot \cos(i\varphi)\ N\ i$=1 n. In the next step the shape contributions were removed to show the individual fluctuation independent of size and shape.[12] To remove the influence of shape the two first harmonics of the cosine series were subtracted generating $\Delta r(\varphi)'$ from $\Delta r(\varphi)$.

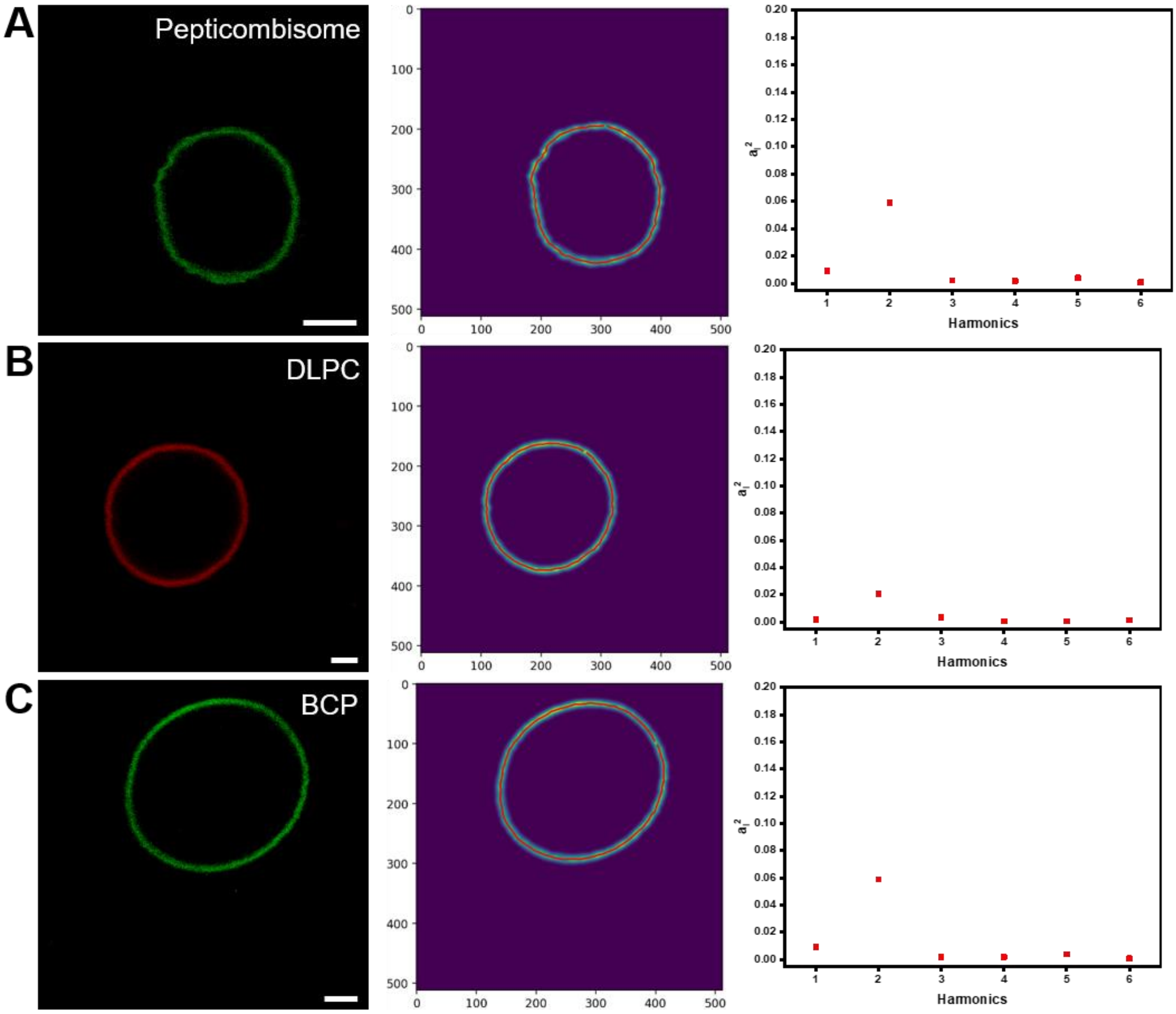

Figure S9. Confocal images of (A) pepticombisome, (B) DLPC liposome, and (C) BD33-b-EO73 BCP (left column). Binary images (central column) and amplitude of the harmonics (right column). Scale bars: 2µm.

2.3 Introduction of functionality

The co-assembly of pepticombs with NTA(Ni) functionalized lipids allowed the immediate selective binding of his-eGFP to the vesicle periphery. Control experiments confirmed that the binding of eGFP can be attributed to the functional lipid, as his-eGFP does not bind to pure pepticombisomes even after 1h of contact (Figure S10).

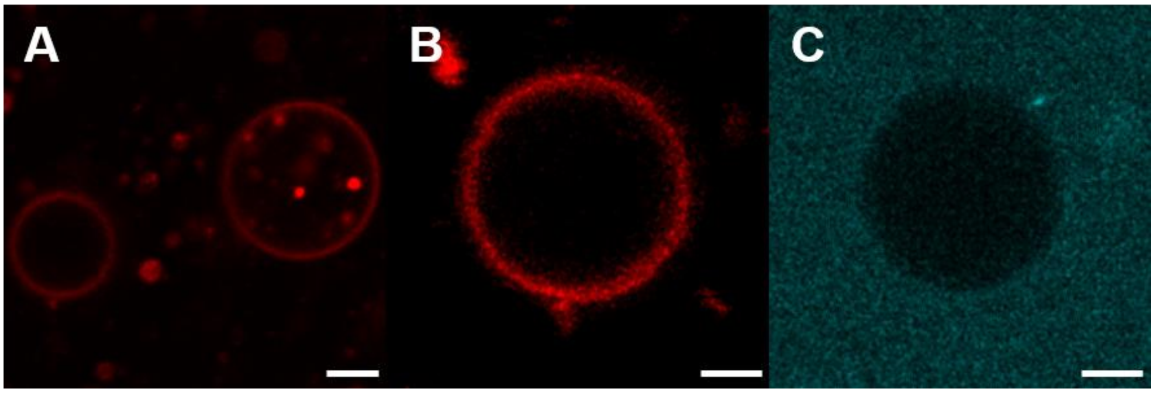

Figure S10. Control experiment of his-eGFP binding to pure pepticombisomes. (A) Pepticombisomes formed by thin film rehydration labeled with Nile Red. The (B) pepticombisome membrane does not show (C) any accumulation of eGFP 1 h after addition. Scale Bars: A: 5µm B,C: 2µm

3. References

[1] C. A. Schneider, W. S. Rasband, K. W. Eliceiri, *Nat. Methods* **2012**, *9*, 671-675.

[2] D. Nečas, P. Klapetek, *Open Phys.* **2012**, *10*, 181-188.

[3] M. J. Abraham, T. Murtola, R. Schulz, S. Páll, J. C. Smith, B. Hess, E. Lindahl, *SoftwareX* **2015**, *1*, 19-25.

[4] G. Bussi, D. Donadio, M. Parrinello, *J Chem Phys* **2007**, *126*, 014101.

[5] S. Grimme, J. Antony, S. Ehrlich, H. Krieg, *J. Chem. Phys.* **2010**, *132*, 154104.

[6] B. Hess, *J. Chem. Theory Comput.* **2008**, *4*, 116-122.

[7] G. A. Kaminski, R. A. Friesner, J. Tirado-Rives, W. L. Jorgensen, *J. Phys. Chem. B* **2001**, *105*, 6474-6487.

[8] H. J. C. Berendsen, J. R. Grigera, T. P. Straatsma, *J. Phys. Chem.* **2002**, *91*, 6269-6271.

[9] a) J. D. Hunter, *Comput. Sci. Eng.* **2007**, *9*, 90-95; b) C. R. Harris, K. J. Millman, S. J. van der Walt, R. Gommers, P. Virtanen, D. Cournapeau, E. Wieser, J. Taylor, S. Berg, N. J. Smith, R. Kern, M. Picus, S. Hoyer, M. H. van Kerkwijk, M. Brett, A. Haldane, J. F. Del Rio, M. Wiebe, P. Peterson, P. Gerard-Marchant, K. Sheppard, T. Reddy, W. Weckesser, H. Abbasi, C. Gohlke, T. E. Oliphant, *Nature* **2020**, *585*, 357-362.

[10] N. Otsu, *IEEE Trans. Syst. Man. Cybern.* **1979**, *9*, 62-66.

[11] H. A. Faizi, C. J. Reeves, V. N. Georgiev, P. M. Vlahovska, R. Dimova, *Soft matter* **2020**, *16*, 8996-9001.

[12] a) H. P. Duwe, E. Sackmann, *Phys. A* **1990**, *163*, 410-428; b) R. Dimova, *Adv. Colloid Interface Sci.* **2014**, *208*, 225-234.
